# The effects of analytical and experiential rumination on autobiographical memory specificity in individuals with a history of major depression

**DOI:** 10.1016/j.brat.2007.05.009

**Published:** 2007-12

**Authors:** Catherine Crane, Thorsten Barnhofer, Claire Visser, Helen Nightingale, J. Mark G. Williams

**Affiliations:** Department of Psychiatry, University of Oxford, Warneford Hospital, Oxford OX3 7JX, UK

**Keywords:** Autobiographical memory, Depression, Mood, Rumination, Recovery, Retrieval

## Abstract

This study examined the relationship between analytical rumination and autobiographical memory specificity in participants with a history of depression. Participants completed the autobiographical memory test twice, once before and once after an 8 min manipulation designed to increase either an abstract/analytical or concrete/experiential mode of information processing. Results indicated a significant three-way *time* (pre, post)×*manipulation* (analytical, experiential)×*depressive rumination* (high, low) interaction. This interaction was the result of a significant decline in memory specificity from pre- to post-manipulation in individuals reporting high levels of rumination about symptoms when depressed who were allocated to the analytical condition. The findings of this study extend previous work, suggesting that low memory specificity in formerly depressed patients may be a function of state levels of analytical self-focus, with this cognitive style being more easily reinstated in the recovery phase in those who report a greater trait tendency to ruminate about symptoms when low in mood.

## Introduction

A difficulty in retrieving specific autobiographical memories has been repeatedly demonstrated in patients suffering from depression (e.g. Kuyken & Dalgleish, 1995; Puffet, Jehin-Marchot, Timsit-Berthier, & Timsit, 1991; Moore, Watts, & Williams, 1988; Williams & Scott, 1988). This deficit is of interest because it has been linked to poor problem-solving skills in depressed and suicidal patients (Evans, Williams, O’Loughlin, & Howells, 1992; Pollock & Williams, 2001; Sidley, Whitaker, Calam, & Wells, 1997), the persistence of depressive symptoms in patients diagnosed with major depressive disorders (i.e. Brittlebank, Scott, Williams, & Ferrier, 1993; Peeters, Wessel, Merckelbach, & Boon-Vermeeren, 2003) and the presence of summer depressive symptoms in patients with seasonal affective disorders (Dalgleish, Spinks, Yiend, & Kuyken, 2001). Further, there is evidence to suggest that at least in some samples over-general memory persists following recovery (i.e. Nandrino, Pezard, Poste, Reveillere, & Beaune, 2002; Spinhoven et al., 2006), and may be linked to a subsequent risk of recurrence of depressive episodes (Mackinger, Loschin, & Leibetseder, 2000; Mackinger et al., 2003, but see Spinhoven et al., 2006 for a failure to replicate).

A recent review of the research literature on memory specificity in individuals experiencing emotional disorder has outlined three interacting mechanisms that appear to contribute to low memory specificity and its associated processes (Williams et al., 2007)—deficits in executive capacity, the experience of trauma and consequent avoidance, and a tendency to become captured by analytical self-focused thinking (e.g. depressive rumination). The study reported here focuses on this third contributory process—the association between an analytical mode of information processing and low memory specificity.

A number of studies have explored the effects of manipulations, which increase or decrease analytical self-focused thinking on memory specificity in patients who are depressed or dysphoric. These have demonstrated that short-term manipulations that reduce analytical thinking temporarily ameliorate low memory specificity (e.g. Watkins & Teasdale, 2001, 2004; Watkins, Teasdale, & Williams, 2000), relative to manipulations that maintain analytical thinking. These studies further suggest that although depressive rumination is predominantly self-focused, it is not the self-focus per se that leads to low specificity but rather the mode of self-focused information processing adopted, whether this rumination is abstract and analytical or concrete and experiential (e.g. whether individuals abstract from and analyse their experiences or focus directly on these experiences from moment to moment, Watkins & Teasdale, 2001).

There is clear evidence of low memory specificity in currently depressed patients, but the findings for recovered patients with a history of depression are more mixed. While some studies have identified persisting deficits in memory specificity (i.e. Spinhoven et al., 2006), others have found levels of specificity more similar to healthy controls (Crane, Barnhofer, & Williams, 2007; Williams, Barnhofer, Crane, & Beck, 2005). One possibility is that while executive capacity deficits normalise on recovery, the tendency to engage in analytical self-focused thinking may continue to contribute to low specificity, but only in certain contexts or under certain circumstances. From this perspective, it is assumed that analytical rumination has both state and trait components. Individuals may differ in the ease with which an analytical mode of processing can be activated (contributing to their trait tendency towards this form of rumination), but the level of analytical rumination at any given time (state level) may be dependent on immediate contextual factors. Thus, impaired retrieval of specific autobiographical memories may be observed in susceptible recovered depressed participants where the context encourages increases in analytical thinking.

The aim of this study was to directly examine the effects of increasing or decreasing an analytical mode of information processing on level of memory specificity in formerly depressed patients who were currently well. It was hypothesised that participants who reported engaging in high levels of analytical rumination when depressed (high trait tendency to ruminate) would show reduced memory specificity when given an induction designed to increase levels of analytical thinking (state effect), while those low in rumination allocated to the analytical condition and those high and low in rumination allocated to an experiential induction would not show such a decrease.

## Method

### Participants

Participants were recruited from the community through posters and announcements on a local community website. Volunteers were recruited who had been depressed in the past but were now well, and those who expressed an interest were given further information about the study. In total, 34 individuals were deemed eligible for inclusion. The final sample included 11 males and 23 females with a mean age of 31 years (SD=14 years). The majority of the sample was Caucasian (1 participant was British Asian and 1 Asian) and well educated, with 71% having participated in higher education.

All participants met criteria for one or more past episode of major depression and all were in recovery at the time of participation. Thirteen participants (38% of the sample) had experienced suicidal ideation when depressed and a further 10 (29%) had made one or more suicide attempts (*n*=5 with two or more attempts). The median number of prior depressive episodes was 3. Eight participants had experienced one previous episode of depression, six had experienced two, nine had experienced three, one had experienced four and nine had experienced five or more episodes (with five of these people reporting chronic symptoms or episodes too numerous to count reliably). Six participants (18%) had received inpatient treatment for depression and six were taking antidepressant medication at the time of participation in the study. Two participants had current mild social phobia, one current mild OCD, four alcohol dependence or alcohol dependence in partial remission, one bulimia, one generalised anxiety disorder and two borderline personality disorder.

### Procedure

Participants attended the Department of Psychiatry for two sessions. The first comprised a clinical interview and the completion of several questionnaires. Approximately 1 week later, participants returned to complete several cognitive tasks (those of relevance to the current study are described below). Participants were randomly allocated to one of four groups (with AMT word sets, A/B, and type of manipulation, abstract/experiential, counterbalanced across groups).^1^ Participants began by completing several practice items on the autobiographical memory test. Following this, they were presented with the first 18 cue words. Participants were then asked to complete the 8 min experiential or analytical attention manipulation (described below). The experimenter remained in the room with the participant but did not interact with them. Following the manipulation period, participants completed a second set of 18 AMT cues. Mood was rated on VAS scales immediately before and after the experimental manipulation. No other tasks were completed between the manipulation and AMT testing. Participants were fully debriefed and paid £20 for participating in the study. The study received ethical approval from the University of Oxford, Central University Research Ethics Committee.

### Measures and materials

#### Clinical interview

Recovery was assessed using an interview based on the NIMH recovery criteria (Keller, Shapiro, Lavori, & Wolfe, 1982). Recovery was defined as no more than 1 week of minimal symptoms of depression in the past 8 weeks. Participants were also interviewed using the borderline personality disorder module of the Structured Clinical Interview for DSM (SCID)-II (First, Gibbon, Spitzer, & Williams, 1997), and the Mini International Neuropsychiatric Interview (MINI; Sheehan et al., 1998) in order to assess the presence of prior major depression, suicidality, and other current or past axis I disorders. The MINI is a structured interview that derives DSM-IV diagnoses and has been validated against the SCID-III-R (Sheehan et al., 1998). It has the advantage of being quicker to administer than the SCID, making it more appropriate for use in experimental studies. Interviews were conducted by C.C., a post-doctoral research psychologist. Audiotapes of a random sample of eight interviews were reviewed by an experienced clinical psychologist who was instructed to note any inconsistencies between the symptoms reported by participants and the decisions reached by the interviewer. Diagnoses derived by both raters were consistent in all cases.

#### Attention manipulations (Watkins & Teasdale, 2004)

Participants were randomly allocated to receive a manipulation designed to increase either an analytical (abstract rumination) or an experiential (concrete rumination; see footnote 1) mode of information processing. In both cases, participants were asked to read through a series of 28 cards that contained identical self-relevant statements (for example, “the physical sensations in your body”, “the way you react”, “how hopeful or hopeless you are feeling”, “your present feelings of fatigue or energy”), preceded by one of two introductions.

In the analytical condition, participants were asked to “think about” the topic in the statement (e.g. “Think about your present feelings of fatigue or energy”). This was preceded by the instruction “as you read these items use your imagination and concentration to think about the causes, meanings, and consequences of the items”. In the experiential condition participants were asked to “focus your attention on your experience of” the topic in the statement (e.g. “focus your attention on your present feelings of fatigue or energy”). This was preceded by the instruction “as you read the items, use your imagination and concentration to focus your mind on each experience. Spend a few moments visualising and concentrating on your experience, attempting to find a phrase, image or set of words that best describes the quality of what you sense”. Thus, the experiential instructions encouraged participants to focus on direct experiences, while the analytical instructions encouraged participants to abstract from and analyse the experiences. Two previous studies have demonstrated that both manipulations leave the state of mood unaffected (Watkins & Moulds, 2005; Watkins & Teasdale, 2004), and both produce increases in self-focus (Watkins & Moulds, 2005) or result in equivalent levels of post-manipulation self-focus (Watkins & Teasdale, 2004). Further, in currently depressed individuals, the experiential manipulation improves interpersonal problem solving and reduces over-general autobiographical memory relative to analytical manipulation (Watkins & Moulds, 2005; Watkins & Teasdale, 2004).

#### *Autobiographical memory test* (Williams and Broadbent, 1986)

Participants were presented with 2 sets of 18 cue words (1 pre- and 1 post-manipulation, 9 positive and 9 negative trait adjectives in each set). The word sets were matched for frequency, emotionality and imageability using data from the University of Western Australia Psycholinguistic Database. The words in set A were *strong, lazy, untidy, interesting, stupid, reliable, boring, poor, kind, calm, failure, sad, responsible, anxious, optimistic, fat, confident* and *happy*. The words in set B were *friendly, energetic, angry, humorous, loving, mean, rich, lonely, pessimistic, carefree, selfish, tidy, jealous, independent, aggressive, weak, understanding* and *bitter*. Participants were instructed to recall a specific memory in response to each cue word. ‘Specific’ was defined as a memory of an event that occurred at a particular time and place and lasted no longer than 1 day. Participants were also instructed not to repeat memories or to describe memories of events occurring in the last 12 months. Examples were given and participants completed 3 practice items. In the test phase participants were given 30 s per cue.

All responses were audio-taped and scored for level of specificity by the experimenter as specific (4), extended (3), categoric (2), semantic associations (1) or omissions (0). A second rater, blind to all other details of the experiment, rated a sample of 235 memory responses. The weighted *κ* based on scoring of memories (4-3-2-1-0 as above) indicated excellent agreement, *κ*=0.82. Previous research (e.g. Crane et al., 2007) has indicated that in formerly depressed community samples there may be relatively low rates of different types of non-specific memory, precluding analyses of different types of error (e.g. number or proportion of categoric memories). We therefore followed Williams et al. (2006) and used total specificity scores (summed scores where specific memories are assigned a score of 4, extended memories a score of 3, categoric memories a score of 2, associates a score of 1 and omissions a score of 0), pre- and post-manipulation, as the main outcome measures in the analyses. These scores are based on the assumption, derived from hierarchical models of memory retrieval (such as the Self-Memory System model of Conway & Pleydell-Pearce, 2000), that memory responses of different types differ systematically in the level of specificity from specific memories at the top to semantic associates and then omissions as the least specific type of response. While this represents an approximation, it enables information on different classes of memory error to be incorporated into the dependent variable in samples where absolute levels of each type of error are small. This measure was used to improve sensitivity to changes in the level of memory specificity, but we also report (in footnote 3) comparable results for analyses based on number of specific memories retrieved.

#### Response-style questionnaire—Ruminative Responses Scale (Treynor, Gonzalez, & Nolen-Hoeksema, 2003).

This questionnaire contains three sub-scales assessing different aspects of rumination—*depression-related rumination* (analytical rumination focused on the symptoms of depression, 12 items, e.g. “think about feelings of achiness and fatigue”, “think about how hard it is to concentrate”), *brooding* (described by the authors as “moody pondering”, e.g. “think ‘why can’t I handle things better?’”) and *reflection* (items referring to neutrally valenced pondering, e.g. “analyse recent events to try and understand why you are depressed”). Since items ask participants how often they think about and analyse different experiences, the ruminative responses scale (RRS), broadly speaking, assesses levels of analytical rumination. Some studies have focused on the total RRS score as a measure of depressive rumination while others have favoured specific sub-scales. For the purposes of the present study, we were most interested in the *depressive rumination sub*-*scale* of this questionnaire because the items used in the manipulations of analytical and experiential thinking (e.g. Watkins & Teasdale, 2004) are based on the content of this sub-scale. Hence, it was assumed that priming and reinstatement of analytical rumination by manipulation would be most likely to occur in those in whom this symptom-focused depressive rumination had been a habitual tendency during past depressive episodes.

Previous research has suggested that the depressive rumination sub-scale may be confounded with severity of depression (e.g. Treynor et al., 2003). However, in the current sample there was no significant correlation between endorsement of items on the depressive rumination sub-scale items and (a) number of depressive symptoms reported during the worst episode of major depression, *r*_s_=0.09, *p*>0.60, (b) current level of depression as assessed by the Beck Depression Inventory (BDI), *r*_s_=−0.12, *p*=0.50, or (c) number of prior depressive episodes, *r*_s_=0.18, *p*=0.32. Although this increases confidence that the depressive rumination measure was independent of severity of depression participants had experienced, for comparability with other studies the analyses were repeated using the total RRS scale and are reported in footnote 4.

#### Beck Depression Inventory (BDI-II, Beck, Steer and Brown, 1996)

The BDI-II is a well-established measure of depressive symptomatology. It contains 21 groups of statements, which assess the severity of symptoms of depression over the preceding 2 weeks.

#### Current mood ratings

To ensure that any effects of the manipulations of mode of processing were not simply a consequence of changes in mood, participants completed visual analogue scales to rate levels of happiness and despondency prior to and following the manipulation. Two 10 cm scales were presented to participants, each labelled from ‘not at all’ sad/happy to ‘extremely’ sad/happy. Participants were asked to make a mark on each scale to indicate their mood ‘at this moment’.

## Results

### Statistics

An *α* level of 0.05 was used for all statistical tests. Analysis was conducted using SPSS version 14.^2^

### Participants

In total, 16 participants were allocated to the analytical and 18 to the experiential manipulations, with 5 males in the analytical and 6 in the experiential condition. One of the male participants (who was allocated to the analytical condition) was identified as a statistical outlier on the pre-manipulation memory specificity score and was excluded from further analysis as he retrieved only one specific memory and it was unclear whether he had understood the task instructions. Mean levels of depressive rumination were somewhat higher in women than in men but there was no significant difference between groups, *F* (1, 31)=1.12, *p*=0.30. Two Spearman's rank order correlations were computed to explore the association between number of prior depressive episodes (1, 2, 3, 4, 5+) and specificity of memory (pre- and post-manipulation). These indicated no significant associations (memory specificity score: pre, *r*_s_=0.15, *p*=0.40; post, *r*_s_=0.10, *p*=0.56). There was also no significant effect of gender on pre- or post-manipulation specificity scores and no significant main effects or interactions for gender when it was entered into the analysis (*males*: pre: *M*=59.00, SD=11.21, post: *M*=56.90, SD=15.37; *females*: pre: *M*=61.17, SD=8.22, post: *M*=61.21, SD=8.52). Analyses were therefore collapsed across gender.

### Current mood

All participants were in recovery from depression, reporting no more than 1 week of minimal symptoms of depression in the preceding 8 weeks. The mean BDI-II score of the sample was low at *M*=3.73 (SD=3.97). Of the 33 participants, 32 reported no more than minimal symptoms of depression on the BDI-II (BDI-II score <13) and one participant scored in the mild symptoms range (14–19). There was no significant difference in BDI-II score between participants allocated to the analytical and experiential rumination conditions, *U*=133.50, *Z*=−0.37, *p*=0.71.

### Effect of manipulations on mood

VAS happiness and despondency ratings were taken immediately pre and post the analytical and experiential manipulations (happiness: *analytical* pre: *M*=5.55, SD=1.73, post: *M*=5.48, SD=2.01; *experiential* pre: *M*=5.11, SD=1.84, post: *M*=5.41, SD=1.23; despondency: *analytical* pre: *M*=2.75, SD=1.77, post: *M*=2.92, SD=2.11; *experiential* pre: *M*=2.67, SD=1.83, post: *M*=2.97, SD=1.87). Separate 2 (*time*: pre, post) by 2 (manipulation: *analytical*, *experiential*) mixed ANOVAs were computed to examine the effects of the manipulations on mood. These identified no significant main effects, or *time×manipulation* interactions (all *F*'s<1), confirming that the two manipulations had minimal and equivalent effects on participants’ mood state.

### Autobiographical memory

The mean specificity score for participants in each condition and the descriptive statistics for participants’ responses to the AMT are shown in Table 1. The majority of participants’ responses were specific and where participants did not give a specific response their errors fell relatively equally across the different ‘error’ categories, with very low mean numbers of memory response in each category, precluding analysis of different types of error.

### Effects of manipulations on memory specificity

We divided the sample into two groups based on a median split on the depressive rumination sub-scale of the RSS (low depressive rumination, *n*=17; high depressive rumination, *n*=16). A 2 (*time*; pre-test, post-test) by 2 (*manipulation*; analytical, experiential) by 2 (*rumination*: high, low) mixed ANOVA was conducted to examine the effects of the manipulation and level of rumination on memory specificity. This analysis revealed no significant main effects of time or manipulation, and no significant two-way interactions. However, there was a significant three-way *time×manipulation×depressive rumination* interaction, *F* (1, 29)=7.23, *p*=0.012, *η*_p_^2^=0.20.

**Fig. 1:**
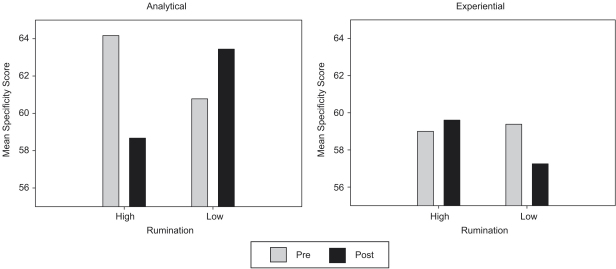
Effects of manipulations to increase analytical and experiential rumination on autobiographical memory specificity in formerly depressed participants reporting high and low levels of rumination when depressed.

Bonferroni-corrected post-hoc comparisons were conducted to explore the origins of this three-way interaction. First, specificity scores were compared between those allocated to the analytical and experiential rumination conditions, at both pre-test and post-test, separately for those high and low in rumination. There were no significant effects (all *p*'s>0.39). Second, specificity scores were compared between participants high and low in rumination, at both pre-test and post-test, separately for those allocated to the analytical and experiential manipulations. Again there were no significant effects (all *p*'s>0.2). Finally, changes in memory specificity from pre-test to post-test were examined separately for those high and low in rumination, allocated to the analytical and experiential manipulation conditions. These analyses indicated a significant reduction in memory specificity score from pre-test to post-test among those allocated to the analytical rumination condition who were high in depressive rumination, *M_i−j_*=−5.50, SE=2.23, *p*=0.025. There were no other significant effects (all *p*'s>0.30).^3,4^ The effects of the manipulations on memory specificity in high and low ruminators are shown in Fig. 1.

## Discussion

### Overview

This study explored the effects of manipulations designed to increase or decrease state levels of analytical self-focused thinking on autobiographical memory specificity in a recovered depressed sample. The results of the study indicated that induction of an analytical mode of information processing resulted in reduced memory specificity, but only in those previously depressed individuals who reported high trait tendencies towards depressive rumination.

A recent review of the literature on low memory specificity and emotional disorder has suggested that three processes—analytical rumination (and capture by self-discrepancies), deficits in executive capacity and functional avoidance—are likely to contribute to the phenomenon in depressed samples (Williams et al., 2007). It is suggested that for some individuals reduced memory specificity develops initially to serve an affect regulation function, reducing the negative affect that would otherwise occur in response to the retrieval of specific distressing mental content. However, although reduced specificity may be negatively reinforced as a result, like other safety behaviours, it has significant deleterious consequences for the individual (e.g. impairing interpersonal problem solving and reciprocally reinforcing and being reinforced by ruminative tendencies), which ultimately increase the likelihood of persistent depression (see Williams et al., 2007 for further discussion). It is therefore of interest to understand more about the processes that maintain low memory specificity during depression and those that may encourage it to persist or re-emerge in the recovery phase.

While low specificity is a robust observation in currently depressed samples, the relative contribution of executive capacity deficits, functional avoidance and rumination to the phenomenon is likely to differ from person to person. Not all individuals who are depressed engage in high levels of rumination and not all have experienced traumatic events. Since deficits in executive capacity remit on recovery from depression the relative importance of rumination and functional avoidance in contributing to low specificity in participants who have recovered from depression is likely to be greater. This study suggests that state factors that promote an analytical mode of information processing may lead to reduced memory specificity in the recovery phase in a sub-set of formerly depressed patients—those who have high trait tendencies towards analytical rumination and in whom specific contextual factors are therefore able to reinstate this habitual processing style. Low memory specificity in the recovery phase may be less observable in those patients in whom lack of specificity during depressive episodes is primarily attributable to deficits in executive capacity and control and may be elicited by different factors in those for whom functional avoidance is the key contributory process.

Previous studies have differed in the extent to which low memory specificity has been observed in formerly depressed patients. The findings of this study suggest that one possibility is that studies have also differed in the extent to which the testing situation has had the capacity to increase state levels of analytical thinking. For example, studies that test memory specificity immediately after a detailed clinical interview or in the hospital where the individual received treatment for depression may be more likely to reinstate rumination and reduce memory specificity than studies that do not include these elements. Additionally, different studies may have tended to sample from sub-populations of formerly depressed patients differing in trait ruminative tendencies. Community studies requiring a sustained period of recovery following a depressive episode may be less likely to include high ruminators since rumination increases the severity of depressive symptoms and is related to the emergence of depressive symptoms in response to stressful events (e.g. Nolen-Hoeksema, 2000; Nolen-Hoeksema & Morrow, 1991). In contrast, studies that follow up clinic samples into the recovery phase will be less open to this bias. Finally, the cue words themselves may activate an analytical mode if they map onto themes that have been the subject of depressive rumination in the past (e.g. Crane et al., 2007) reducing levels of memory specificity observed.

### Limitations

Although the findings of this study are consistent with theoretical predictions, there are a number of limitations that require consideration. Most importantly, the study utilised a relatively small sample, which might have increased likelihood of type-2 errors, and the findings should therefore be considered with caution until there is evidence of replication.

Second, the analyses focused on the moderating effect of trait levels of depressive rumination on memory specificity. Levels of depressive rumination were explored since it is this type of analytical rumination that maps most closely on to the content of the manipulations of processing style. However, other researchers have suggested that brooding is the most pernicious form of rumination and that measures of depressive rumination may be contaminated with the severity of an individual's depressive illness (e.g. Treynor et al., 2003). In the current sample, we found no significant association between levels of depressive rumination and number of symptoms of depression during the most severe depressive episode, number of depressive episodes or current BDI score. Further, the same results held when the sample was subdivided using a median split on the total RRS score, increasing confidence that the findings are not simply attributable to comparisons between more and less severely affected participants. However, the focus of the current study was on the priming of a mode of information processing (whether analytical or experiential), the capacity to do this in different participant groups and the down-stream consequences of this for memory specificity, rather than on the effects of the particular content to be processed within this mode. It may therefore be interesting in future research to explore whether trait tendencies towards different types of rumination (for example, brooding versus reflection) are more closely related to low memory specificity during depressive episodes or to the reactivation of low specificity in certain contexts following recovery.

Third, in this study participants were requested to retrieve memories of events that had occurred more than 12 months ago. This instruction was included because an additional aim of the current study was to explore biases in the sampling of memories from across the lifespan. However, introducing this restriction may have increased the difficulty of the task faced by participants and may have acted as a cognitive load. Although instructions were constant across experimental conditions it is possible that the effect of the manipulations on memory specificity may have been more pronounced as a result. Such an effect might arise if participants had less cognitive resources available to override the effects of the manipulations, as a result of the extra demand placed on executive capacity. Although we believe that this is unlikely to be a significant factor in the results, work is beginning to explore the effects of cognitive load on memory specificity in recovered depressed samples and this is an interesting line of future research (e.g. Barnhofer, Crane, Spinhoven, & Williams, in press).

Finally, previous studies have demonstrated that analytical and experiential manipulations have equivalent effects on self-focus, leaving mood state unchanged (e.g. Watkins & Teasdale, 2004; Watkins & Moulds, 2005) and the current study also ruled out changes in mood as a possible factor in the results. One previous study (Watkins & Moulds, 2005) has also examined the extent to which the manipulations affected the abstractness of participants’ responses to the task of interest (using the judgments of an independent rater) as a further manipulation check. Since ratings of memory specificity are very close to ratings of memory abstractness, in the current study we explored the effects of the manipulations on this variable directly, without the inclusion of a further manipulation check. However, it would clearly be beneficial in future research to develop a task that could be used to independently and indirectly assess the effects of these manipulations on mode of processing, since the effects are likely to be subtle and not necessarily available to self-report.

### Conclusions

Previous research has indicated that analytical self-focused thinking contributes to the maintenance of low memory specificity in currently dysphoric individuals (Watkins & Teasdale, 2001, 2004). This study demonstrates that such a thinking style also appears to reduce levels of memory specificity in individuals who are in recovery and asymptomatic, but that this effect occurs only in those for whom analytical rumination about symptoms of depression is a more habitual pattern. These findings are of relevance with regard to the question of why low memory specificity is a more inconsistent feature in previously depressed individuals and they illustrate potential ways in which effects of analytical thinking may cascade into further vulnerability processes increasing the risk for relapse. These findings are also interesting from a clinical perspective. Previous research has suggested that an intervention (mindfulness-based cognitive therapy, MBCT) that aims to reduce the risk for depressive relapse in part by increasing patients’ capacity to disengage from analytical rumination, also increases memory specificity (e.g. Williams, Segal, Teasdale, & Soulsby, 2000). The findings of the current study support the suggestion that the reduction of habitual tendencies to ruminate may represent one plausible mechanism of action of MBCT on memory specificity and suggest that modifying trait tendencies to ruminate may have important consequences for other aspects of psychological functioning.

## Figures and Tables

**Table 1:** Descriptive data for memory responses given by participants allocated to the analytical and experiential conditions, pre and post the manipulation of thinking style

Type of memory	Analytical		Experiential	
	Pre	Post	Pre	Post
Specific	13.13 (4.60)	12.69 (5.26)	13.06 (3.50)	12.78 (4.45)
Extended	1.06 (1.57)	1.25 (1.61)	1.44 (1.62)	1.50 (1.54)
Categoric	1.38 (1.75)	1.50 (2.37)	1.17 (1.65)	1.22 (1.77)
Semantic associate	0.69 (1.54)	1.19 (2.23)	0.28 (0.57)	0.50 (0.86)
Omission	1.69 (1.58)	1.38 (1.78)	2.06 (2.46)	2.00 (2.63)
Total specificity score	62.13 (7.51)	61.53 (8.13)	59.17 (10.26)	58.56 (12.68)

## Footnotes

1 AMT word set order (A/B or B/A) and experimental manipulation (analytical or experiential) were counterbalanced across participants such that participants could be allocated to one of four groups. At the start of the experiment, the final number of participants to be recruited was unknown. Therefore, randomisation was blocked such that within each group of four participants recruited one would be allocated to each experimental condition. Randomisation envelopes were prepared and a member of the research team blind to clinical information randomised each participant following the clinical interview.

2 The memory specificity scores at pre- and post-test had mild negative skewness. However, when the main analyses were repeated with the memory specificity scores root transformed (to normalise the data) this yielded materially equivalent results, and for clarity the analyses on the untransformed data are reported.

3 These analyses were repeated using the number of specific memories retrieved, rather than the total specificity score, as the dependent variable. The significant three-way *time*×*rumination group*×*manipulation* interaction remained, *F* (1, 29)=5.89, *p*=0.02. Post-hoc tests again indicated a significant reduction in number of specific memories retrieved from pre- to post-induction in high ruminators allocated to the analytical manipulation, *M_i_*_−_*_j_*=−0.22, SE=1.06, *p*=0.05. There were no other significant main effects or interactions, and no other significant post-hoc comparisons.

4 These analyses were also repeated dividing the sample into two groups on the basis of a median split on the total RRS score (low rumination=18, high rumination=15). Again there was a significant *time*×*rumination group*×*manipulation* interaction, *F* (1, 29)=4.75, *p*=0.04, and a significant reduction in specificity in the high ruminators allocated to the analytical manipulation from pre- to post-manipulation, *M_i−j_*=5.50, SE=2.37, *p*=0.03. There were no other significant main effects or interactions, and no other significant post-hoc comparisons.
